# Nutrition and Altitude: Strategies to Enhance Adaptation, Improve Performance and Maintain Health: A Narrative Review

**DOI:** 10.1007/s40279-019-01159-w

**Published:** 2019-11-06

**Authors:** Trent Stellingwerff, Peter Peeling, Laura A. Garvican-Lewis, Rebecca Hall, Anu E. Koivisto, Ida A. Heikura, Louise M. Burke

**Affiliations:** 1 Canadian Sport Institute-Pacific, Institute for Sport Excellence, 4371 Interurban Road, Victoria, BC V9E 2C5 Canada; 2 Department of Exercise Science, Physical and Health Education, University of Victoria, Victoria, BC Canada; 3 School of Human Sciences (Exercise and Sport Science), University of Western Australia, Crawley, Australia; 4 Western Australian Institute of Sport, Mt Claremont, Australia; 5 Australian Institute of Sport, Bruce, ACT Australia; 6 Mary Mackillop Institute for Health Research, Australian Catholic University, Melbourne, VIC Australia; 7 School of Health and Sports Sciences, University of the Sunshine Coast, Sippy Downs, QLD Australia; 8 Norwegian Olympic Sports Centre, Norwegian Olympic and Paralympic Committee and Confederation of Sports, Oslo, Norway

## Abstract

Training at low to moderate altitudes (~ 1600–2400 m) is a common approach used by endurance athletes to provide a distinctive environmental stressor to augment training stimulus in the anticipation of increasing subsequent altitude- and sea-level-based performance. Despite some scientific progress being made on the impact of various nutrition-related changes in physiology and associated interventions at mountaineering altitudes (> 3000 m), the impact of nutrition and/or supplements on further optimization of these hypoxic adaptations at low–moderate altitudes is only an emerging topic. Within this narrative review we have highlighted six major themes involving nutrition: altered energy availability, iron, carbohydrate, hydration, antioxidant requirements and various performance supplements. Of these issues, emerging data suggest that particular attention be given to the potential risk for poor energy availability and increased iron requirements at the altitudes typical of elite athlete training (~ 1600–2400 m) to interfere with optimal adaptations. Furthermore, the safest way to address the possible increase in oxidative stress associated with altitude exposure is via the consumption of antioxidant-rich foods rather than high-dose antioxidant supplements. Meanwhile, many other important questions regarding nutrition and altitude training remain to be answered. At the elite level of sport where the differences between winning and losing are incredibly small, the strategic use of nutritional interventions to enhance the adaptations to altitude training provides an important consideration in the search for optimal performance.

## Key Points

**Table:** 

While the effects of high altitude on the endocrine systems, energy intake, resting metabolic rate and body mass are severe, it appears that resting metabolic rate is also increased, albeit to a smaller extent, at low to moderate altitudes, and targeting adequate energy intake is important for optimizing health and appears to be an emerging factor associated with optimizing altitude adaptations.
Despite being iron-replete, a blunted erythropoietic response is observed in non-iron supplemented athletes during simulated altitude, with data demonstrating that most athletes will maximize the hypoxia-induced increases in hemoglobin mass while consuming ~ 100–200 mg of elemental iron daily in oral form, with most evidence coming from iron salts.
There is insufficient evidence to recommend high-dose single antioxidant supplementation to attenuate altitude-induced oxidative stress, as this may actually impair endurance and altitude-based training adaptations; although this does not seem to occur with the integration of ample amounts of antioxidant-rich *foods* into athletes’ daily diets.

## Introduction

Altitude training is a common feature of elite endurance preparation and is a strategically periodized intervention in various elite athlete programs [1–4]. As extensively researched, the primary adaptive responses athletes seek during altitude sojourns include primarily the erythropoietin (EPO)-driven increase in red blood cells (or hemoglobin mass (HBmass) [1–5]). Although under-studied in elite athlete populations, there are also important non-hematological altitude adaptations, such as increased buffering capacity and potential improvements in exercise economy, as well as the extensive genetic responses of hypoxia inducible factor 1-alpha (HIF) [6]. Furthermore, recent work has also elucidated the optimal training altitudes (~ 1600–2400 m [5]) for elite athletes and theoretical timing of exposure (~ 2–4 weeks) and training prior to competition [7]. [Note: all altitudes referenced below will use the thresholds defined by Bartsch and Saltin [8]: “near sea level” (0–500 m); “low altitude” (500–2000 m); “moderate altitude” (2000–3000 m); “high altitude” (3000–5500 m); and “extreme altitude” (> 5500 m).]

**Fig. 1:**
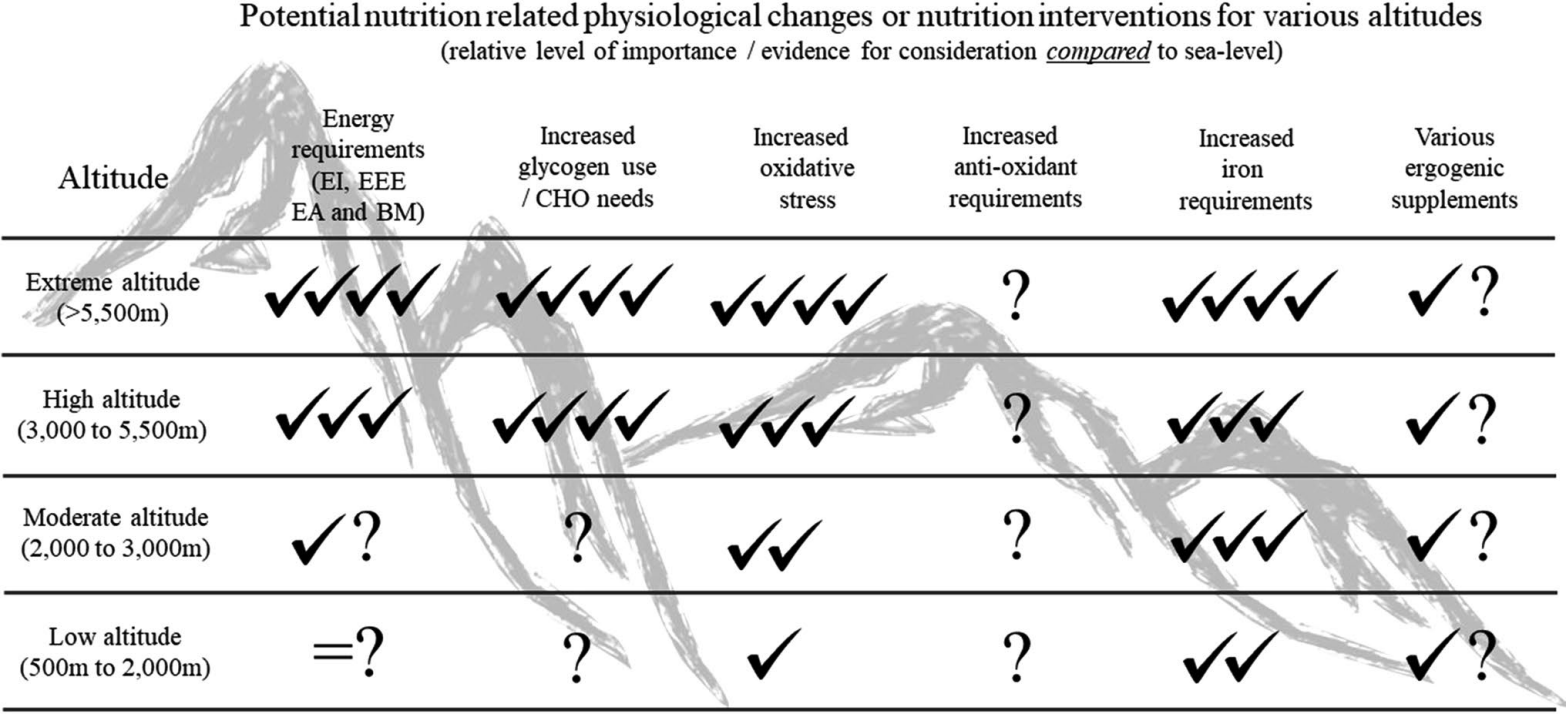
Potential nutrition-related physiological/metabolic changes or nutrition interventions for various altitudes. Altitude cut-offs are as established by Bartsch and Saltin [8]. All rankings are reflective of the relative level of importance and impact, and/or evidence, compared to sea level. An equal (=) *sign* represents equivalent evidence and importance as at sea level. ✓✓✓✓ is convincing evidence, ✓✓✓ is strong evidence, ✓✓ is moderate evidence, and ✓ is low or emerging evidence for a physiological/metabolic change or for nutrition intervention consideration. *?* indicates emerging evidence or potentially theoretical rationale, but no published studies at specific altitude or requires more scientific confirmation. *BM* body mass, *CHO* carbohydrate, *EA* energy availability, *EEE* exercise energy expenditure, *EI* energy intake

Although “nutrition at altitude” has been commonly reviewed [9–15], the vast majority of nutrition recommendations are based on research conducted at high to extreme altitudes, which do not correspond to the training altitudes typical of elite athletes (~ 1600–2400 m). However, recently, several new publications on nutrition interventions at low–moderate altitudes have emerged. Within this narrative review, we have highlighted six major nutrition intervention themes to consider at low–moderate altitudes, including whether there are altered energy, iron, carbohydrate (CHO), hydration and/or antioxidant requirements, in addition to considerations for the potential use of various ergogenic aids. Figure 1 highlights these six nutrition themes, the general strength of recommendations, and gaps in knowledge (Table 1), across the various altitudes. Although there are many approaches to implementing hypoxia (see review by Millet et al. [16]), this current review will primarily focus, unless otherwise indicated, on nutrition interventions for natural terrestrial altitude training. Where possible, we will contrast our knowledge of metabolism and nutrition from high–extreme altitude research to that of low–moderate altitudes, and identify appropriate future research directions.

**Table 1:** Equivocal data and future research directions examining the efficacy of nutrition interventions at low to moderate altitude (~ 1600–2400 m) across various nutrition themes

Altitude nutrition theme	Equivocal data and future research directions
General macronutrient and energy requirements (e.g., EI, EEE, EA, RMR and BM)	Substantiate the consistency and quantity of potential altitude-induced shifts in EA, RMR or BM at low to moderate altitudes (~ 1600–2400 m) [32, 38, 39]
	Does long-term RED-S compromise baseline pre-altitude HBmass and/or iron values? [32]
	Does RED-S compromise within altitude camp HBmass and/or other altitude-specific adaptations (injury/illness)? [30–33]
	Do altitude-induced shifts in RMR and/or appetite actually impact body composition outcomes, or is this just a training camp effect? [38–41]
	Do athletes naturally adjust their dietary energy and macronutrient intake while at altitude? If so, are their dietary routines at altitude in line with current recommendations?
Hydration requirements	Enhanced elucidation of actual low to moderate altitude-induced hydration requirements (especially in dry vs. moist altitude environments)
	What are the actual low to moderate altitude shifts in PV, and can they be attenuated via optimal hydration interventions?
	Does reduced hydration PV status, over time, potentially impact upon cardiac outputs, training quality and/or health status?
Glycogen/CHO and protein utilization changes	Are there actual shifts at low to moderate altitudes (~ 1600–2400 m) towards increased CHO metabolism and protein turnover at the same relative exercise intensities as sea level?
	Do athletes need to appreciably increase dietary CHO and/or CHO fueling during training sessions at low–moderate altitudes? [9–15]
Increased oxidative stress and anti-oxidant requirements	Is there an appreciable increase in RONS at low–moderate altitudes that is linked to injury/illness and/or altitude-induced adaptation?
	What is the impact of single-source high-dose antioxidant supplementation on altitude-induced training adaptations?
	Are there individual situations at altitude/in hypoxia where diets high in antioxidants are especially warranted to prevent illness?
	What degree of oxidative stress is necessary to foster the adaptive response of altitude training, and when does oxidative stress become detrimental (e.g., increased inflammation, delayed recovery)?
Increased iron requirements	Are low baseline ferritins, with optimal hemoglobin, contraindicated for altitude training camps when iron supplementation will be optimized? [11, 54, 55]
	What are the lowest iron and/or hemoglobin values that would contraindicate attending an altitude camp?
	Are morning single-daily-dose iron protocols more effective than late-night single-dosing protocols at altitude as compared to alternating-day supplementation protocols? [57, 60]
	Are iron salts or heme-based iron supplements most effective? Which supplements produce the least GI issues in athletes (given absence of GI issues is a prerequisite for good compliance)?
	What are the iron requirements for sustained (many months) low–moderate altitude sojourns?
	Will an intervention iron dose–response study (including low, moderate and high iron doses) result in significant HBmass differences and/or result in potential negative effects/downsides of excess free iron? Will it contribute to higher oxidative stress?
Various ergogenic supplements	Substantiate or refute the mechanistic and/or performance outcomes of key identified IOC supplements [107] at all altitudes (Fig. 1)
	Further elucidate the adaptive hypoxic training response of buffers and/or nitrates at altitude, or whether they actually prove to attenuate altitude adaptations [120]
	Further data to support or refute altitude-based supplements such as NAC or *Ginkgo biloba* and whether vitamins B_6_, B_12_ and D and/or branched amino acids or glutamine impact on altitude adaptations [151, 152, 157, 159]
	Investigate whether acute nitrate supplementation prior to key altitude sessions, in contrast to chronic nitrate supplementation, affects the adaptive response to altitude and performance (potentially allows higher speed/watts at key training sessions?)

Key associated references for further reading are included

*BM* body mass, *CHO* carbohydrate, *EA* energy availability, *EEE* exercise energy expenditure, *EI* energy intake, *GI* gastro-intestinal, *HBmass* hemoglobin mass, *IOC* International Olympic Committee, *NAC N*-acetylcysteine, *PV* plasma volume, *RED-S* relative energy deficiency in sport, *RMR* resting metabolic rate, *RONS* reactive oxygen and nitrogen species

## Macronutrient and Hydration Considerations at Altitude

Changes in macronutrient and fluid needs of athletes undertaking altitude training should be considered from two perspectives: (1) the direct effects of a hypoxic environment on physiological processes that affect metabolism, and daily utilization/loss of these nutrients; and (2) indirect effects due to a change in training load. Although the focus of this review is on the former perspective, it should be briefly observed that many athletes deliberately or inadvertently alter their typical internal and external training loads during an “altitude camp” [4, 17, 18]. Although a reduction in external training load may temporarily occur due to fatigue associated with the additional physiological stress, this may not represent a reduced internal training load, due to the augmented hypoxic stress. Furthermore, many athletes consider altitude per se, or an environment away from their home commitments, to present an opportunity for intensified training or weight loss. Although some aspects of such approaches [e.g., low energy availability (EA)] will be further discussed (and discouraged) in this review, the scientist should not neglect the importance and effects of training underpinning fuel, fluid and protein requirements, as observed with conventional sea-level training.

Most studies of the specific effects of altitude on nutrient needs have been conducted at high to extreme altitudes; these have described downregulation of protein synthesis and increased protein requirements [19] as well as changes in energy, CHO and fluid turnover [20]. Although these altitude-induced changes in metabolism have been less well studied at the typical low–moderate training altitudes, subtle effects are likely and may become amplified when they interact with high training volumes and the numerous camps and durations of altitude used by some elite athletes. Shifts towards greater CHO utilization have sometimes been shown at high altitudes [21], although a study in females at 4300 m showed decreased CHO utilization [22]. Despite high-altitude inconsistencies in changes in CHO oxidation, most suggest increasing dietary CHO requirements to replace muscle glycogen stress and a greater need/benefit of CHO intake during exercise [9, 13]. However, until systematic study of these concepts during the types of training undertaken by elite athletes at low–moderate altitudes is undertaken, guidelines to address this remain speculative. Manipulating CHO availability according to the goal of the training sessions (e.g., training with low CHO availability to drive cellular adaptation and high CHO availability to promote performance and training intensity) is another tool available to athletes to optimize training outcomes [23]. Further investigation is needed of protocols to implement and achieve these CHO availability manipulations and how they might be best optimized within altitude training.

Hypoxia and the low air humidity associated with altitude environments are also likely to increase fluid losses at rest and during training. Local weather conditions can also vary according to the altitude location and time of the year and will also interact with altitude-specific effects. Increased respiratory water loss and the diuresis often seen in the early response to altitude exposure can create a significant increase in water requirements at the same time that reduced thirst and changes in fluid availability in a new environment may alter usual drinking practices [2, 24, 25]. Therefore, athletes should consider altitude training a time of increased risk for dehydration and both monitor and address their hydration status appropriately [e.g., monitor urine characteristics and daily body mass (BM) changes, and be proactive with fluid intake during and after training sessions and with meals]. Taken together, whether there are consistent and performance-relevant changes in hydration and/or CHO and protein oxidation at low–moderate altitudes requires further scientific validation. At this point, we hypothesize that the individual training load via the “training camp effect” and the local altitude camp weather conditions probably influence nutritional recommendations to a greater extent than the potentially more mild hypoxic effects at low–moderate altitudes.

## Hypoxic Effects on Energy Availability, Body Mass and Altitude Adaptations

Adequate EA is an important consideration for both sea-level and altitude training. EA reflects the amount of energy that remains after exercise for use by other body systems, including the endocrine, immune and reproductive systems, and is calculated as energy intake (EI) minus exercise energy expenditure (EEE) relative to fat-free mass (FFM) [26]. Pioneering work by Loucks and colleagues, using controlled laboratory studies, has defined low EA as < 30 kcal/kg FFM/day, below which impairments to reproduction, endocrine function and bone health have been demonstrated [26]. This concept of low EA has recently been termed relative energy deficiency in sport (RED-S) and has multiple implications in both male and female athletes for iron metabolism, injury and illness, training adaptation and performance [27]. As such, optimal EA (~ 45 kcal/kg FFM/day) is essential for long-term health and performance [26, 27]. However, while it is not clear whether low–moderate hypoxic exposure has additive effects on EA requirements, there are several emerging, and compelling, concepts to suggest that EA will play an important role in optimizing hypoxic adaptation.

The suppression of sex hormones (estrogen and/or testosterone) levels due to low EA may impair hematological adaptations to altitude. For example, low EA and iron metabolism are linked [28], which may have direct effects on hematological adaptations at altitude (see Sect. 4.1). Furthermore, just 18 h of fasting in rats exposed to extreme altitude (7000 m) reduced hypoxia-induced EPO production by 85% [29]. Low EA has also been shown to drastically increase the risk of injury and illness [26, 27], which at altitude, has consistently demonstrated deleterious effects on hypoxia-induced increases in HBmass. Indeed, reductions in HBmass following altitude training have been consistently reported in ill/injured athletes [30–33] compared to the typical 3–7% increase in HBmass in healthy counterparts [34]. Meanwhile, estrogen is important for iron homeostasis through its suppression of the peptide hormone hepcidin, which results in an increase in iron bioavailability [35]. Further, testosterone treatment in older males can reverse anemia [36]. This is supported by observed findings that amenorrheic elite female runners had an 8% lower (*p* < 0.05) baseline HBmass when compared to their eumenorrheic counterparts prior to an altitude camp [37].

### Energy Availability Considerations at Low to Moderate Altitudes

While the effects of high-altitude exposure on endocrine systems, EI, resting metabolic rate (RMR) and ultimately BM are consistent and severe, the handful of research findings at low–moderate altitudes are much less consistent and appear to be far less pronounced. Indeed, emerging case-study data include loss of appetite reported by four rowers who reported increased fatigue during a 12-day intense training block at 1800 m [38]. Conversely, data from five elite runners reported an increased appetite, with no change in EI, after 4 weeks of living and training at 2200 m [39]. However, it is important to point out that the rowers increased their training load at altitude by 113% [38], while the runners only increased their training load 37% [39], compared to sea-level training loads. Furthermore, both negative [40, 41] as well as optimal energy balance (EB) [42] have been reported in both elite Kenyan and Ethiopian runners at moderate altitudes. Meanwhile, when 48 elite female and male distance athletes maintained moderate EA (33–36 kcal/kg FFM/day, assessed over a 1-week period) across 3–4 weeks of training at 2150 m, BM remained stable [32, 37]. However, it should be noted that dietary records [43] are poor estimates of EA when used in isolation (Table 1), and the variability of these BM and EA outcomes demonstrate that more research is required.

With respect to RMR, to our knowledge, only two studies have investigated the effects of moderate altitude on this variable in elite athletes. The first study observed five elite runners for 4 weeks at 2200 m, reporting an increased RMR, by 19% [39]. In comparison, the second study followed four elite rowers, who reported no change in RMR after 12 days at 1800 m [38]. Collectively, this work presents the notion that when EA is adequate (as indicated via no change in BM in the study by Woods et al. [39]), it appears that RMR is increased at moderate altitudes similarly, albeit to a smaller extent, to high/extreme altitudes. However, given the small participant populations used here, more research is required to confirm these findings that RMR is increased at low–moderate altitudes (Table 1).

**Fig. 2:**
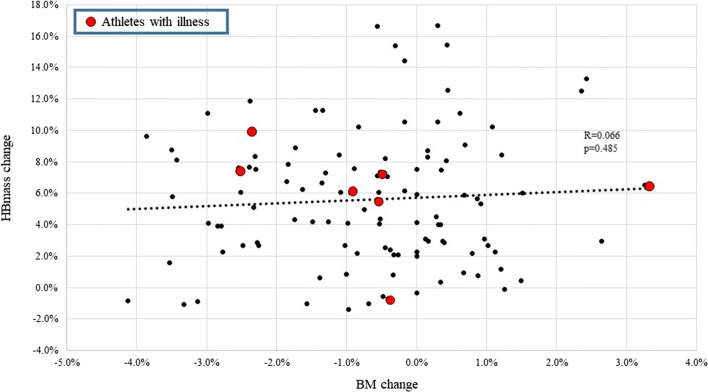
The relationship between the pre- to post-altitude camp (~ 3–4 weeks) percentage change in HBmass and the pre- to post-altitude camp percentage change in BM across 114 different unique athlete observations over 3–4 weeks of camp duration from 2015 to 2018 (unpublished observations). Athletes highlighted as a red dot had illness throughout the camp. The dashed line represents a linear regression (*R* = 0.066; *p* = 0.485). *BM* body mass, *HBmass* hemoglobin mass

In terms of changes in BM during altitude training camps (~ 3 weeks), studies report no change [32, 39, 40, 42, 44] or minor BM decreases [38, 41, 45] when exposed to moderate altitudes. The decreases in BM may reflect low EA and have been associated with negative EB [41] and stable RMR [38]. Meanwhile, when BM was maintained (suggesting optimal EA), stable hormone concentrations [32] and increased RMR [39] were noted across an altitude camp. The importance of maintaining BM via optimal EA is highlighted by studies showing that a failure to do so may negatively influence hematological adaptations to altitude. For example, McLean et al. [33] reported that football players who lost ≥ 2 kg BM during training at 2100 m only increased HBmass by 2.5% as compared to 5.0% in those who maintained BM. Furthermore, elite male cyclists significantly lost BM (− 1.2 kg) and FFM (− 1.0 kg) while failing to increase HBmass over a 31-day altitude camp, likely due to overtraining and/or illness [45]. Conversely, unpublished observations from four separate altitude training camps (of ~ 3–4 weeks duration, from 2015 to 2018) with the same HBmass procedure/laboratory (Hypo2, Flagstaff, AZ, USA), featuring 114 observations, demonstrated a − 0.6 ± 1.5% BM decrease and a 5.6 ± 4.1% increase in HBmass, with no relationship between changes in HBmass and BM reported, and no relationship with illness (Fig. 2). However, changes in BM alone are a poor indicator of EA status, as prolonged and/or severe reductions in EI may lead to adaptive thermogenesis, which promotes maintenance or gain of BM despite low EA [46]. It is also important to note that upon arrival to altitude there is a contraction of plasma volume (PV) [47, 48] and typical altitude associated dehydration. Accordingly, acute small weight loss (i.e., < 2% BM) should not be confused with an actual reduced EA, as BM alone is a poor indicator of EA. Indeed, loss of body water due to increased ventilation and diuresis is an essential short-term adaptation to altitude which serves to increase arterial oxygen content via increased hemoglobin concentration prior to longer-term erythropoietic adaptation [49]. Residual BM loss associated with PV contraction is typically reversed upon return to sea level [31]. Overall, the impact that training at moderate altitudes has on BM, EA, and subsequent endocrine and metabolic (e.g., RMR) effects warrants further investigation (Table 1).

## Micronutrient Considerations to Optimize Adaptation to Altitude

Unless there are clinical deficiencies or allergy/intolerance-dictated specific diets, athletes do not have unique, or elevated, vitamin and mineral requirements at sea level compared to the general population [50]. However, hypoxia provides a distinct environment where several micronutrients may need to be considered. This section will focus on the impact of iron status and dietary supplements, deliberating on whether anti-oxidant supplementation is warranted to minimize the production of reactive oxygen and nitrogen species (RONS) and oxidative damage at low–moderate altitudes.

### Iron and Altitude

There are several factors that impact an athlete’s HBmass response at altitude, including the hypoxic dose (~ + 1% increase per 100 h at ~ 2000 m [34, 51]) and baseline HBmass [32, 52]. Beyond this, the nutrition intervention receiving the most scientific attention with regards to optimizing adaptations to moderate altitudes is the mineral iron. In 1992, investigators such as Benjamin Levine, James Stray-Gunderson and colleagues were amongst the first to highlight that pre-existing iron deficiency (serum ferritin of 15 ± 3 vs. 69 ± 10 μg/l) without iron supplementation at 2500 m of altitude compromised the red blood cell adaptive response to altitude training [53]. Accordingly, pre-altitude ferritin cut-offs of < 30 ng/ml and < 40 ng/ml have often been used as a pre-altitude “check” to ensure optimal adaptations and/or whether to supplement iron in females and males, respectively [11]. However, these pre-altitude ferritin cut-offs, in combination with iron supplementation, have not been scientifically validated (Table 1), although they have been utilized in a recent altitude study that showed expected HBmass increase after 3 weeks at moderate altitude (+ 4.7% [44]). Furthermore, anecdotally, athletes who have low pre-altitude ferritin (> 15 but < 30 ng/ml) with normal pre-altitude hemoglobin, but who are supplemented with iron throughout an altitude camp, appear to still exhibit optimal HBmass adaptations. Indeed, several studies have shown no relationship between pre-altitude ferritin stores and the magnitude of the HBmass response [44, 54, 55]. To add to this, re-analysis of data from 49 elite athletes training at 2100 m while consuming ~ 100–200 mg of elemental iron daily also supports no relationship between pre-altitude ferritin and subsequent HBmass responses, as long as athletes supplement with iron throughout the altitude camp (Pearson correlation between baseline ferritin and percentage change in HBmass = − 0.1296, *p* = 0.38 [32]).

**Fig. 3:**
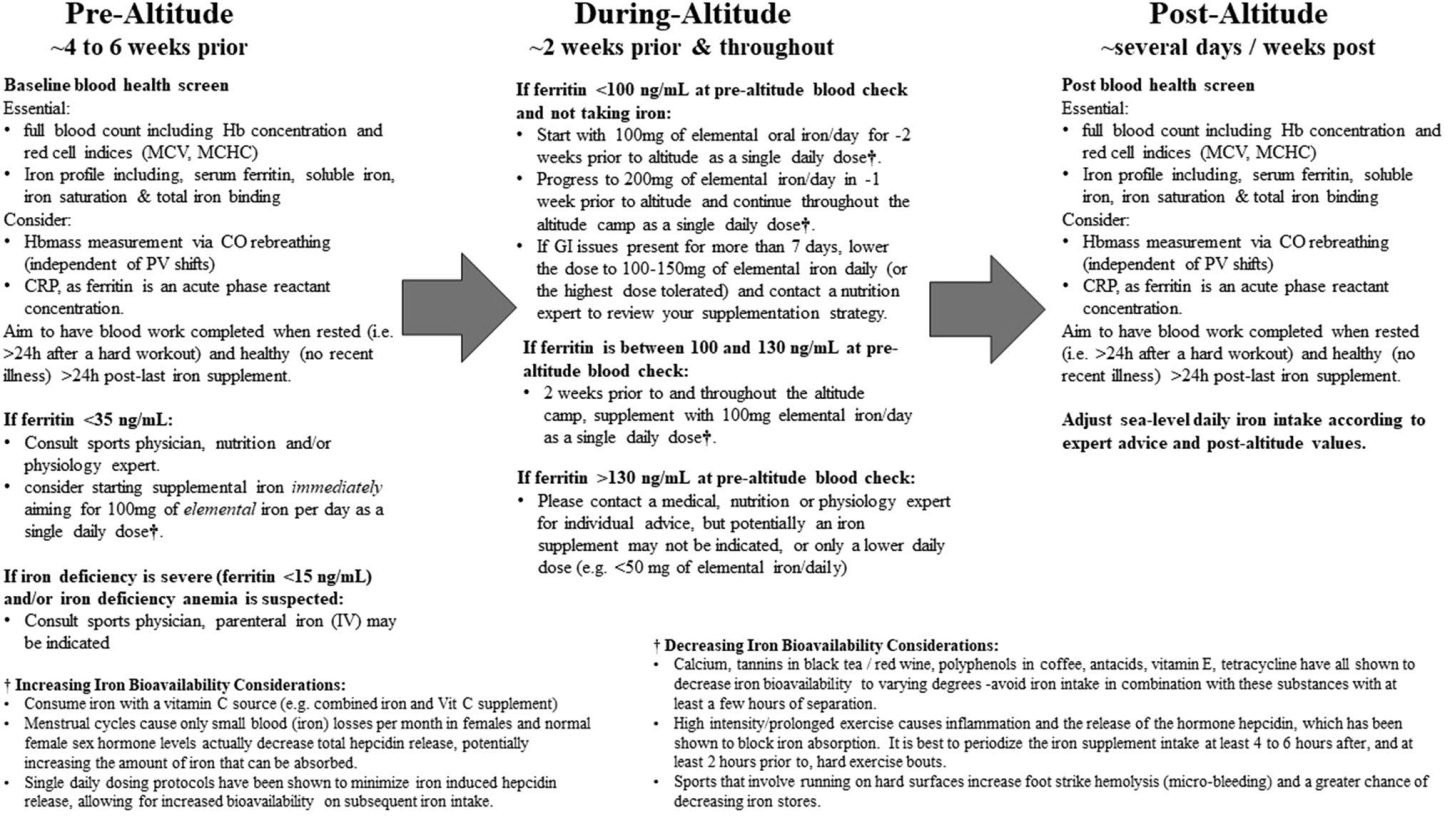
Contemporary blood health screening and supplemental iron recommendations before, during and after altitude. Recommendations are based on the following references [11, 56, 57, 60, 64, 163–166] and are not to replace local and/or national sport expert advice and policies, and do not constituent medical advice. Final recommendations should always be sought from a sports medicine physician. *CO* carbon monoxide, *CRP* C-reactive protein, *GI* gastro-intestinal, *Hb* hemoglobin, *HBmass* hemoglobin mass, *h* hours, *IV* intravenous, *MCHC* mean corpuscular hemoglobin concentration, *MCV* mean corpuscular volume, *PV* plasma volume, *Vit* vitamin

Current recommendations are to assess iron status 8–10 weeks prior to altitude training [11] and to commence oral supplementation 2–3 weeks prior to altitude exposure, and to continue this supplementation throughout (Fig. 3 [55–57]). However, iron stores can change appreciably in 8–10 weeks [58, 59], and our current recommendation is to aim for pre-altitude blood work ~ 4–6 weeks prior to allow for more precise pre-altitude ferritin assessment, yet time to still supplement and correct prior to altitude if required (Fig. 3). Regarding the optimal iron dose at low–moderate altitudes, retrospective analysis of hematological data collected from athletes (*n* = 178) engaged in altitude training at moderate altitudes (1350–3000 m) demonstrated greater HBmass increases in iron-supplemented athletes versus those who were not supplemented [60]. In this study, athletes who did not supplement with iron had HBmass increases of only 1.2%, while athletes who supplemented with 105 mg or 210 mg had HBmass increases of 3.3% and 4.0%, respectively. Our altitude iron recommendations established on ferritin cut-offs of < 100, ~ 100–130 and > 130 ng/ml (Fig. 3) are based on interpolation and/or extrapolation of existing data [56, 57, 60]. Accordingly, a blunted erythropoietic response was also observed in non–iron-supplemented athletes during simulated live-high train-low (LHTL) despite being iron replete [55]. Furthermore, altitude studies that have supplemented ~ 200 mg of elemental iron per day have only shown modest increases in pre- to post-ferritin levels (~ 5–30% [57, 60]), indicating the increased iron utilization at altitude and the low risk for iron overload. Nevertheless, these ferritin cut-offs require further scientific validation, as no definitive iron dose–response study at low–moderate altitudes in athletes currently exists.

Taken together, current evidence suggests that most athletes will maximize the hypoxia-induced increases in HBmass while consuming ~ 100–200 mg of elemental iron daily in oral form, with most evidence relating to iron salts. Recent advances in intravenous (IV) iron formulations have radically changed the accessibility and safety associated with IV iron delivery [61], raising questions surrounding its suitability and efficacy as a supplementation option during altitude exposure. However, IV iron did not further augment the HBmass response to 3 weeks of simulated altitude training compared with standard oral supplementation practices (105 or 210 mg of elemental iron/daily) in non-anemic, trained endurance athletes [55]. Thus, it would appear that oral iron supplementation remains the most appropriate option for iron-replete individuals, which better aligns with the “no needle” policies of many sporting organizations’ governance processes.

The regulation of the peptide hormone hepcidin needs to be considered when looking to maximize iron bioavailability in hypoxia. Hepcidin is often referred to as the “master iron regulator,” as increased hepcidin causes a decrease in iron absorption and iron recycling within the body [62]. Hepcidin is suppressed in hypoxic conditions [57, 63], but is upregulated when high amounts of iron are present in the circulation and subsequent to exercise training [64], which, in turn, reduces iron availability since both dietary iron uptake from the gut and expression of iron on macrophages are impaired [65]. Accordingly, a number of iron dosing protocols are possible, such as single or split daily doses, or dosing every other day [66, 67]. Interestingly, at sea level, multiple daily doses of iron caused an increase in hepcidin, and a decrease in the percent of iron absorbed from subsequent iron doses in iron deficient females [66], suggesting single dosing protocols may be superior. However, despite elevated hepcidin and decreased bioavailability (% relative absorbed) with two doses per day [66], or daily iron dosing compared to alternate days [67], the total iron absorbed remains greater with a higher frequency of supplementation [notwithstanding any individual gastro-intestinal (GI) issues with greater individual doses].

Further support for a single daily iron dosing protocol comes from a recent applied study in elite runners over a training camp at 2100 m [57]. This study compared a split (100 mg elemental iron at 7–8 AM plus 100 mg between 9 and 10 PM) versus a single (200 mg elemental iron at 9–10 PM) equivalent dose of ferrous fumarate daily for ~ 3 weeks. While both supplemented groups experienced a significant increase in HBmass post altitude, the single-dose group had a significantly greater increase (6.7 ± 6.3%; *p *= 0.048) compared to the split-dose group (4.6 ± 3.9%). The trade-off may be greater reports of GI concerns with the single dose, as there was a 37% increase in the overall GI distress score associated with the single versus split dose over the first 2 weeks. However, this difference was not apparent by week 3, suggesting gut adaptation can occur to the greater single dose [57]. Also, there may be different GI tolerance to different types of oral iron supplements [68], which also may be individually trialed. Of note, the efficacy of an alternate-day iron supplementation protocol is yet to be explored in athletes at altitude, but offers a promising possible avenue for further research (Table 1).

In conclusion, since no correlation has been observed between pre-altitude ferritin stores and the magnitude of the erythropoietic/HBmass response [32, 54, 55], it appears that iron availability via supplementation during altitude is more important for optimal adaptations than pre-altitude iron stores [56, 57]. Nevertheless, optimizing iron bioavailability, via optimal iron dose timing, requires an appreciation of the temporal effects of hepcidin that are influenced by baseline ferritin [64], timing of multiple daily iron doses [66, 67], timing, duration and intensity of training [64], and diurnal effects [69]. Figure 3 highlights our current knowledge and recommendations regarding blood health screening and supplemental iron recommendations before, during and after altitude, including highlighting factors that will increase or decrease oral iron bioavailability. We recommend the involvement of a sports medicine physician in this process, as excess iron supplementation and clinically elevated endogenous iron stores can have negative health consequences [70, 71].

### Antioxidant Considerations

Exercise at moderate altitudes is associated with increased production of RONS with reduced antioxidant capacity, leading to oxidative stress [72, 73]. The excessive overproduction of RONS, in excess of the endogenous antioxidant defense systems, can cause damage to lipids, proteins and DNA which may impair cell and immune function, resulting in delayed post-exercise recovery [74]. Both acute [75, 76] and chronic exposure to hypoxia [72, 77, 78] augments oxidative stress in well-trained athletes, while reduced antioxidant capacity may persist for up to 2 weeks following altitude training [79]. Interestingly, normobaric hypoxia appears to produce a larger increase in oxidative stress than hypobaric hypoxia [80], while a recent study in a team sport setting showed no impact of intermittent hypoxia on biomarkers of oxidative stress [81]. Although several factors can modulate the oxidative stress response to altitude (e.g., the intensity and type of training [82]), it can be generalized that a greater level of hypoxic stimulus results in greater oxidative stress (Fig. 1 [83, 84]). The clinical implications of altitude-induced oxidative stress are not entirely clear [85, 86], beyond being linked to acute mountain sickness (AMS) at high altitudes [87, 88]. At moderate altitudes, some studies have shown increased inflammation and illness in association with higher levels of oxidative stress [89, 90], but others have not [81]. It is important to note that, although there is some evidence of immunological biomarker disturbances at low–moderate altitudes in elite athletes [91, 92] and anecdotally there is an assumption of increased rate of illness at altitude, there is actually limited evidence of an increased rate of illness at low–moderate altitudes. In fact, a recent athlete and immune function review by Walsh et al. [93] highlights that there is no evidence that exercising in extreme environments poses any additional immune threat, and some recent evidence suggests that immune health may actually be enhanced by regular intermittent environmental stressors.

Given that exogenous antioxidants neutralize free radicals, it is logical to hypothesize that antioxidant supplementation would be a worthy intervention to combat altitude-induced oxidative stress and its potentially associated perils. Although early investigations have shown that antioxidant supplements had modulating effects on oxidative stress and AMS symptoms at high altitudes [94, 95], more recent studies indicate no effect [96–98] or mixed results [99]. Importantly, the majority of the studies have been conducted in military training settings and/or at altitudes > 4000 m, and thus, do not reflect the contemporary altitude training regimes of athletes. Furthermore, none of these studies have assessed the impact of antioxidant supplementation on training adaptation. With the current understanding of the essential role of RONS in initiating the positive adaptive response to endurance training [100], hypoxia [101] and upregulation of the endogenous antioxidant defenses [102], dampening RONS with antioxidants might actually be counterproductive and reduce the adaptive responses to altitude training (Table 1), which has been shown at sea level [103–105]. Two recent studies have examined the effect of antioxidants from food sources on the adaptive response to altitude training [44], and oxidative stress and inflammation [106]. The first study [44] revealed that more than doubling the daily intake of antioxidant-rich foods during a 3-week altitude camp (2320 m) did not interfere with the training responses in elite endurance athletes [measured as HBmass and maximum rate of oxygen consumption (*V*O_2max_)]. While the follow-up study showed that the food-based antioxidant intervention elevated plasma antioxidant capacity and attenuated some of the altitude-induced increases in systemic inflammatory biomarkers, it had no impact on altitude-induced oxidative stress in the elite athlete population [106].

Collectively, there is not sufficient evidence to recommend high-dose single antioxidant supplementation to attenuate altitude-induced oxidative stress, especially at low–moderate altitudes. Furthermore, the impact of antioxidant supplements on the adaptive training response to altitude requires further research (Table 1). At present, integrating ample amounts of antioxidant-rich foods into athletes’ daily dietary regimes while training at altitude seems the most reasonable advice, including a vitamin C source (e.g., orange juice, low-dose vitamin C supplement) with the athletes’ iron supplement to optimize bioavailability (Fig. 3).

## Ergogenic Supplement Considerations During Altitude

Despite a growing body of evidence for a handful of specific ergogenic aids for performance enhancement at sea level [107], there are very few acute or chronic supplementation studies completed in hypoxic conditions. Given that hypoxia changes oxygen extraction, delivery and uptake, as well as altering lactate kinetics and buffering, we would caution against the indiscriminate use of sea-level ergogenic aids until more hypoxia-based data are generated. Nevertheless, there are some preliminary data on nitrate supplementation and some theoretical use of buffers at altitude, along with several other emerging supplements that will be covered in this section.

### Nitrates/Beetroot at Altitude

Nitric oxide (NO) is a pleiotropic signaling molecule and a regulator of many physiological and adaptive processes that are endogenously stimulated by hypoxia. Therefore, dietary nitrate (NO_3_; an NO precursor) supplementation, usually in the form of concentrated beetroot consumption, during hypoxia has garnered much recent attention [108, 109]. Accordingly, one might hypothesize even greater effects of NO_3_^−^ supplementation at altitude. Similar to the data found in normoxia, there are several studies confirming enhanced exercise economy (reduced steady-state *V*O_2_), by ~ 5–10%, in hypoxia and/or enhanced performance outcomes in recreationally active subjects after NO_3_^−^ supplementation [110–112]. However, also similar to the normoxic literature, four recent hypoxia-based studies have failed to show these outcomes in elite endurance trained subjects (*V*O_2max_ > 60 ml/kg/min); although some individuals appeared to benefit [113–117], this is not always the case [118]. Therefore, similar to the normoxic data, the majority of studies in elite endurance-trained subjects show no further benefits of NO_3_^−^ supplementation in hypoxia.

Perhaps not surprisingly, given that NO_3_^−^ supplementation increases O_2_ delivery, in both recreational [111, 112] and elite [116] subjects, NO_3_^−^ supplementation has resulted in a small 1–4% increase in arterial O_2_ saturation (S_a_O_2_) via pulse oximetry during hypoxic exercise (thus less desaturation). However, given the links between decreases in S_a_O_2_ and EPO release [119] and HIF-1-alpha responses, one might question whether chronic NO_3_^−^ supplementation while training at altitude might actually attenuate some of the hypoxia-induced adaptations. Indeed, several studies have shown no performance enhancing or training adaptation effect of chronic NO_3_^−^ supplementation during hypoxic training over 5–6 weeks [120, 121], suggesting that chronic NO_3_^−^ intake might attenuate training adaptations by decreasing the drop in arterial (S_a_O_2_) and muscle O_2_ saturations—factors that serve as “signals” for hypoxic adaptations [120]. Taken together, given the lack of consistent data and/or several studies suggesting contraindications [120, 122], we cannot conclusively recommend NO_3_^−^ supplementation during altitude training in elite athletes unless individual outcomes have been quantified.

### Hypoxic Acid/Base Regulation and Exogenous Buffering Considerations

During progressively intense exercise, the drop in muscular pH via hydrogen ion (H^+^) production, which is exacerbated by hypoxia at the same absolute workloads, has been shown to negatively affect metabolic processes, such as the inhibition of glycolysis and muscle contraction processes, ultimately resulting in fatigue and decreased performance [123]. Therefore, the enhancement of both intra- (inside) and extra-muscular (outside) buffering of H^+^ should lead to an increase in performance where metabolic acidosis is a limiting factor. Accordingly, humans have evolved to have many varying endogenous mechanisms contributing to total buffering capacity, which are innately enhanced upon ascent to altitude [6] and potentially increased by several nutrition-based ergogenic aids. These ergogenic aids with significant *sea-level* evidence [107] include (1) β-alanine (BA) supplementation leading to intra-cellular muscle carnosine synthesis (3–6 g BA/daily for 6–8 weeks) and (2) sodium bicarbonate (NaHCO_3_) or citrate supplementation leading to extra-cellular increases in bicarbonate (HCO_3_^−^) [acutely, ~ 300 mg NaHCO_3_/kg BM taken 1–2 h prior to competition]. It is beyond the scope of this review to unravel the complexities of anaerobic performance determinants and associated potential ergogenic aids (for recent reviews, see Peeling et al. [107] and Stellingwerff et al. [124, 125], as well as the extensive literature on acid–base regulation during exercise [123] and/or during hypoxia [126]). Instead, this section will briefly overview the buffering changes upon ascent to altitude and then examine the scant data on whether exogenous buffering supplements should be considered at altitude (Fig. 1).

Upon immediate ascent to altitude, there is a very rapid hyperventilatory response to hypoxia to raise PO_2_, which leads to respiratory alkalosis, resulting in decreased H^+^, increased pH and increased renal HCO_3_^−^ excretion, resulting in decreased blood HCO_3_^−^; for reviews see Gore et al. [6] and Cerretelli and Samaja [126]. Subsequent to this immediate response, chronic altitude (days to weeks) actually increases intra- and extra-cellular buffering capacity [127–129]. Indeed, hypoxia-induced changes in blood pH can occur in elite 400-m runners in just several days [130]. In fact, enhancement of critical buffering transporters in blood erythrocytes (via increased monocarboxylate and bicarbonate (Cl^−^/HCO_3_^−^) transporters [127]) and a 5–6% increase in muscle buffering [128, 129] have both been shown in just 2 weeks of hypoxia. Therefore, given the drop in blood HCO_3_^−^, one might hypothesize that exogenous NaHCO_3_ or citrate supplementation may actually be more advantageous for performance in hypoxia. However, in the handful of studies that have used NaHCO_3_ or citrate in hypoxic conditions, six studies have shown no ergogenic effect [131–136], while one study has shown an increase in the anaerobic (*W*′) component, which would be suggestive of a performance enhancement, at simulated altitude (14.5% O_2_; ~ 2800 m [137]). Regardless, it is difficult to compare any of these studies, because of different levels of hypoxia, performance protocols and states of hypoxic adaptation, as well as several of the studies being potentially under-powered to detect a performance difference (*n* ≤ 7 [131, 134, 135]). Obviously, much more research is required to better understand the limiting effects of performance in hypoxia, the time course and impact of these extracellular buffering changes in elite athletes at low–moderate altitudes, as well as the mechanism(s) responsible for the subsequent enhanced muscle buffering capacity (Table 1).

The chronic utilization of nutritional buffers to potentially enhance training adaptations is not well understood. For example, there are only a few studies examining the chronic effect of NaHCO_3_ supplementation (5–7 days) in normoxia, all of which have initially demonstrated promising outcomes [138–141]. However, some individuals will suffer from GI upset and/or increased fluid retention and BM increases (+ 1–3% BM) due to the high sodium ingestion [142, 143] following NaHCO_3_^−^ supplementation, potentially negating the performance outcomes [144–146]. All of these negative side effects may be exacerbated with prolonged chronic NaHCO_3_^−^ protocols. Additionally, two studies have examined whether augmented carnosine via BA supplementation may lead to an enhanced training effect, with one study showing a trend for greater resistance-training volume [147] and another finding no influence of BA to further enhance high-intensity interval training [148]. We are unaware of any prolonged chronic NaHCO_3_ supplementation studies conducted at altitude/hypoxia. Conversely, we are only aware of a single study examining the acute effects of NaHCO_3_ supplementation both prior to and after 5 weeks of BA supplementation. All of these trials were conducted in simulated hypoxia (15.5% O_2_ = ~ 2400 m), in which neither BA nor NaHCO_3_ caused any performance benefits [149]. Obviously, our global understanding of the adaptive effects of buffers is not well understood at either sea level or altitude (Table 1).

Taken together, current evidence would not support the use of exogenous NaHCO_3_ or citrate supplementation to augment acute hypoxic performance. Furthermore, given the remaining questions around the innate magnitude and timing of acid–base/buffering changes in elite athletes at low–moderate altitudes, let alone the potential adaptive impact of chronic exogenous buffers, we also cannot conclusively recommend the chronic use of NaHCO_3_ or citrate supplementation. However, due to BA’s long supplementation period (~ 6–8 weeks) to augment muscle carnosine, one might consider its utilization if the timing were critical for post-altitude competitions, with no current anecdotal evidence to report any apparent negative side effects (unpublished observations).

### Other Potential Supplements: *N*-Acetylcysteine and *Ginkgo biloba*

Limited evidence exists for alternative nutritional supplements that have extensive data as potential ergogenic aids for enhancing adaptation to altitude. For example, we are unaware of any intervention studies investigating the impact of vitamins B_6_, B_12_ and D or glutamine or branched chain amino acids in athletes at low–moderate altitudes. Therefore, whether a higher intake of these vitamins and proteins (in the form of supplements) would have additional benefits has not been investigated and again highlights areas for future research. However, when looking beyond the aforementioned prospects of nitrate and buffers, the thiol-containing compound *N*-acetylcysteine (NAC) seems to show *mechanistic* promise. Previous work has shown that NAC increases circulating free cysteine levels, which, in the presence of increased glutathione demand, can support glutathione synthesis and prevent its depletion [150]. Interactions between NAC, cysteine and glutathione are suggested to act in potentially numerous mechanistic ways that may be beneficial for athlete performance, recovery and adaptation. For instance, NAC ingestion is proposed to result in an anti-oxidant effect that minimizes the oxidative stress and inflammatory response imposed from physical activity [151] (see Sect. 4.2). Furthermore, NAC is proposed to enhance fatigue resistance [152] and improve athletic performance [151]; to enhance immune system function [153], hemodynamics and muscle blood flow [154]; and to modulate EPO production and the hypoxic ventilatory response [155]. Intuitively, each of these mechanisms appears likely to support a positive adaptation to hypoxic environments such as altitude exposure. However, scant literature exists to explore such a prospect in applied athlete settings where an altitude sojourn has occurred. Regardless, the previous literature showing a positive impact of NAC supplementation on these relevant physiological outcomes has generally provided the thiol compound in oral dosages ranging from 600–1200 mg/day for a 5- to 9-day period [151, 154, 155]. However, it should be noted that not all literature supports the positive modulation of NAC on EPO production [156], and when consumed in high doses for prolonged periods of time (i.e., 1200 mg/day for 4 weeks, followed by 2400 mg/day for a further 2 weeks), pro-oxidant, rather than anti-oxidant, effects have been reported [157], which may actually serve to attenuate aerobic adaptations (see Sect. 4.2). With this in mind, further research is clearly required before NAC can be recommended as a useful supplement for use prior to and/or throughout altitude exposure in athletes, with factors such as dose, duration of consumption and the resultant potential for mechanistic promise to convert to enhanced adaptation all needing further clarification.

Any indirect benefits of a supplement that might support an athlete’s immune function are of key interest, since it is well-documented that unaccustomed altitude exposure places an additional burden on the immune system [158]. Maintaining an athlete’s immune function and/or reducing the impact of any altitude-induced illness while under such environmental stress may lead to enhanced overall adaptations. With this in mind, there appears to be potential for the use of the herb extract *G. biloba* (GBE), with proposed mechanisms such as reducing tissue hypoxia, increasing vasodilation and, via its anti-oxidant properties, possibly reducing the incidence of a mild AMS [159], characterized by headache, lightheadedness, fatigue, nausea, and insomnia [160]. Such effects can negatively impact on an athlete’s ability to train, and therefore, their overall adaptation to an altitude exposure may be compromised [161]. Although a promising prospect, recent meta-analyses of GBE shows equivocal outcomes of its impact on AMS prevention (only 57% of included studies showed a positive outcome [159]). Investigations exploring the potential prophylactic nature of GBE have used daily split doses of 80–120 mg, consumed over a 3- to 5-day period both before and/or during the altitude sojourn [160, 162]; however, it should be considered that the majority of these studies have focused on trekkers at high to extreme altitudes, rather than athlete populations at low–moderate altitudes. Therefore, similar to NAC supplementation, more research is needed before confident recommendations on GBE supplementation for reductions in altitude-induced illness and/or AMS symptoms can be made.

## Future Directions and Conclusions

Even though the hypoxic stress of altitudes < 2400 m may seem very minor in comparison with mountaineering altitudes, the extreme training intensities and volumes of elite athletes can be compounded and need to be considered. For example, elite endurance athletes may spend as much as 20–25% of the entire year at altitude through the implementation of many camps [1, 4]. Therefore, despite the fact that RMR might be only increased ~ 300 kcal/day at ~ 2000 m [39] (see Sect. 3), if an athlete spends 3–4 months at altitude, this actually amounts to a ~ 25,000 kcal yearly mismatch. Indeed, future altitude studies need to make compelling efforts to quantify both EA and actual training loads to better elucidate whether training camp outcomes are actually due to the hypoxic stress and not due to altered training and/or EA. Within this narrative review, we have focused on six key altitude-related nutrition themes (Fig. 1), and a repeated thesis is the relative lack of data at the low–moderate altitudes (~ 1600–2400 m) that elite athletes typical utilize for optimal training adaptations [5]. Accordingly, many research questions have been raised (Table 1), with a definitive iron dose–response study at natural altitudes in athletes probably being one of the key current gaps in the literature. In conclusion, given the infinitesimal difference between winning and losing, coupled with the fact that most elite endurance athletes/programs utilize altitude to some degree as part of their preparation, the optimization of nutritional interventions to optimize altitude adaptations is an ever-important performance aspect needing consideration.
